# Carbon dioxide protects simulated driving performance during severe hypoxia

**DOI:** 10.1007/s00421-023-05151-1

**Published:** 2023-03-23

**Authors:** Peter Michael Bloomfield, Hayden Green, James P. Fisher, Nicholas Gant

**Affiliations:** 1 Exercise Neurometabolism Laboratory, University of Auckland, Building 907, 368 Khyber Pass Road, Newmarket, Auckland, 1023 New Zealand; 2 Department of Physiology, Faculty of Medical and Health Sciences, Manaaki Mānawa-The Centre for Heart Research, University of Auckland, Auckland, New Zealand; 3 Centre for Brain Research, University of Auckland, Auckland, New Zealand

**Keywords:** Hypoxia, Carbon dioxide, Driving performance, Functional near-infrared spectroscopy

## Abstract

**Purpose:**

We sought to determine the effect of acute severe hypoxia, with and without concurrent manipulation of carbon dioxide (CO_2_), on complex real-world psychomotor task performance.

**Methods:**

Twenty-one participants completed a 10-min simulated driving task while breathing room air (normoxia) or hypoxic air (P_ET_O_2_ = 45 mmHg) under poikilocapnic, isocapnic, and hypercapnic conditions (P_ET_CO_2_ = not manipulated, clamped at baseline, and clamped at baseline + 10 mmHg, respectively). Driving performance was assessed using a fixed-base motor vehicle simulator. Oxygenation in the frontal cortex was measured using functional near-infrared spectroscopy.

**Results:**

Speed limit exceedances were greater during the poikilocapnic than normoxic, hypercapnic, and isocapnic conditions (mean exceedances: 8, 4, 5, and 7, respectively; all *p* ≤ 0.05 vs poikilocapnic hypoxia). Vehicle speed was greater in the poikilocapnic than normoxic and hypercapnic conditions (mean difference: 0.35 km h^−1^ and 0.67 km h^−1^, respectively). All hypoxic conditions similarly decreased cerebral oxyhaemoglobin and increased deoxyhaemoglobin, compared to normoxic baseline, while total hemoglobin remained unchanged.

**Conclusions:**

These findings demonstrate that supplemental CO_2_ can confer a neuroprotective effect by offsetting impairments in complex psychomotor task performance evoked by severe poikilocapnic hypoxia; however, differences in performance are unlikely to be linked to measurable differences in cerebral oxygenation.

**Supplementary Information:**

The online version contains supplementary material available at 10.1007/s00421-023-05151-1.

## Introduction

The brain has a high energy demand, the majority of which must be met via oxidative metabolism. Consequently, energetically demanding functions are extremely sensitive to reductions in oxygen availability, which results in rapid and substantial declines in cognitive function (McMorris et al. 2017). Such functional impairments can occur at high altitude, where the decline in barometric pressure leads to a reduction in the partial pressure of arterial oxygen (O_2_) and hence cerebral O_2_ availability. The extent of cognitive impairment is typically dependent on the magnitude of the reduction in barometric pressure, O_2_ dissociation, and duration of exposure (McMorris et al. 2017; Shaw et al. 2021). Of note, changes in arterial carbon dioxide (CO_2_) tension can also affect simple cognitive tasks in hypoxia (Friend et al. 2019; Stepanek et al. 2014), but whether the performances of more complex tasks with a greater cognitive load are also attenuated remains unknown.

Arterial CO_2_ is a powerful vasodilator of the cerebral blood vessels, exerting its effect by conversion to H^+^ via carbonic anhydrase (Meldrum and Roughton 1933; Ogoh 2019). Conversely, a reduction in arterial CO_2_ leads to cerebral vasoconstriction and a pronounced decrease in cerebral blood flow (CBF) (Battisti-Charbonney et al. 2011; Kety and Schmidt 1948). Hypoxia per se also causes cerebral vasodilation and increased CBF. However, CBF may be reduced by hyperventilation-mediated reductions in arterial CO_2_ during hypoxia, which causes a vasoconstriction opposing the vasodilatory effects of hypoxia (Ainslie and Poulin 2004; Shapiro et al. 1970), although this is dependent on the severity of the hypoxic stress and ensuing poikilocapnic respiratory response (Ogoh 2015; Ogoh et al. 2013). CO_2_ also impacts the oxyhemoglobin curve in a more fundamental way, by altering the affinity of O_2_ for hemoglobin. Hypocapnia causes a leftward shift in the oxyhemoglobin curve, reducing the O_2_ offload at active tissues; this may cause further reductions in cerebral O_2_ availability (Leacy et al. 2019). Importantly, there is some indication that increasing CO_2_ availability during hypoxia can protect cognitive function (Friend et al. 2019; Stepanek et al. 2014). However, these previous studies used simple cognitive function measures (i.e., rapid number reading, simple- and five-choice reaction times); to the best of our knowledge, the effect of CO_2_ availability on complex task performance during severe hypoxia has not been examined. This is important, because the simple tasks commonly utilized in this area may not be reflective of real-world situations, as they typically assess a single neurocognitive domain within a contrived task. Consequently, we used simulated motor vehicle operation—an ecologically valid complex psychomotor task. Driving encompasses a range of neurocognitive domains, including executive function, attention, and reaction time, within an engaging real-world activity.

The aim of this study was to determine the effect of CO_2_ availability on complex task performance during hypoxia. Accordingly, participants inspired four different gas mixtures (room air, and hypoxic air with CO_2_ concentrations that elicited poikilocapnia, isocapnia, and hypercapnia) during a driving simulator task, while frontal cortex oxyhaemoglobin (HbO) and deoxyhaemoglobin (HbR) were measured using multi-channel fNIRS to quantify cerebral oxygenation under these differing conditions. We hypothesized that poikilocapnic hypoxia would degrade simulated driving performance and impair cerebrovascular function, and that these hyperventilation-induced decrements would be prevented by provision of supplemental CO_2_.

## Methods

### Ethical approval

The study aligned with the Declaration of Helsinki, except for registration in a database. Ethical approval was granted by the University of Auckland Human Participants Ethics Committee (approval number: 2672). All participants signed informed consent prior to taking part.

### Participant characteristics

Twenty-one healthy adults (13 males; 8 females) with a mean age of 25.6 years (range 21–36 years) and a mean driving experience of 8.2 years (range 0.75–19 years) took part in the study. Participants were eligible to participate if they were 18–50 years old, passed a health screening questionnaire, and were in possession of a restricted or full New Zealand driver’s license. These categories of license require a minimum of 6 months driving experience and qualify the holder to drive alone. The age range of 18–50 was selected to avoid potential confounding effects of age on performance measures.

### Experimental protocol

The study employed a randomized, repeated-measures design to investigate the effects of inhaled carbon dioxide on driving simulator performance and neurophysiological responses during periods of acute, severe hypoxia. Participants attended two sessions at the experimental facility. The first session was a familiarization session to introduce participants to the driving task and complete the fNIRS set-up process (described below). The second session was experimental where participants performed a driving simulator task (described below) four times, once while normoxic and thrice while inhaling differing hypoxic gas mixtures. The administration of the gas mixtures was randomized to prevent order effects. Before starting any driving, task participants completed a wash-in period (≥ 3 min) to acclimatize to the hypoxic gas and allow end-tidal values to equilibrate (method described below); the driving task did not begin if equilibrium was not attained. Participants had a normoxic washout period of at least 5 min between driving tasks to allow SpO_2_, breathing rate, and end-tidal gas values to return to baseline values.

### Gas manipulation

Participants inspired 4 different gas mixtures; 1 normoxic (P_ET_O_2_ = unmanipulated) and 3 hypoxic (P_ET_O_2_ = 45 mmHg) mixtures with differing amounts of CO_2_ to create isocapnic hypoxia, hypercapnic hypoxia, and poikilocapnic hypoxia. Normoxic gas was room air; the three hypoxic mixtures were a combination of O_2_, N_2_, and CO_2_. Hypoxia was induced by inhaling gas containing 10% O_2_ and balance N_2_. The gas was delivered from a pressurised cylinder to an oronasal facemask (7450 Series V2™, Hans Rudolph Inc., USA) via an intermediate 120 L Douglas bag and 5 L mixing chamber; this method allows a safe and constant delivery of gas while avoiding large pressure changes (Turner et al. 2015a, b). The mixing chamber was used to clamp end-tidal gases. P_ET_O_2_ was clamped at 45 mmHg by adding 100% N_2_ to the mixing chamber at a low flow rate. P_ET_CO_2_ was clamped at the desired level by adding 100% CO_2_ from a pressurised cylinder at a low flow rate. An investigator maintained the gas concentrations manually. Other than being requested to avoid large variations in breathing frequency, participants’ breathing rate and depth were not manipulated. The order of hypoxic gas administration was single-blinded and randomized to prevent order effects; however, participants were aware of when they were breathing room air or hypoxic gas, due to the difference in humidity between the pressurised and non-pressurised gases.

### Driving simulator

A fixed-base STISIM 300WS driving simulator (Systems Technology Incorporated, USA), used in previous studies by our group (Bloomfield et al. 2021; Green et al. 2019), provided a valid measure of complex real-world task performance. This set-up simulated an automatic transmission vehicle and included accelerator and brake pedals, force-feedback steering wheel, and turn signal lever. The graphics were presented across three LCD monitors (Dell, 21″; total display resolution: 3840 × 1080 pixels) positioned approximately 90 cm from participants. The driving task was identical within and between participants and simulated a moderately built-up cityscape (21 buildings min^−1^), with pedestrians (17 pedestrians min^−1^), vehicular traffic (16 cars min^−1^), intersections, and roadworks. See Supplementary Figure S1A and B (https://doi.org/10.6084/m9.figshare.17059727) for an illustration of the simulator set-up and scenario. An audio navigation system guided each participant through 9 intersections (3 left, 3 right, and 3 straight ahead). The duration of the driving task was dependent on vehicle speed but took approximately 10 min to complete. Driving parameters were extracted and prepared for statistical analysis using custom R scripts. The following variables were extracted: vehicle speed, variability of vehicle speed, lateral deviation, variability of lateral deviation, speed exceedances, lane edge excursions, and the number of cars and pedestrians hit.

### Physiological measures

Volume of expired air ($\dot{V}$_E_), tidal volume, and breathing frequency were measured via an MLT1000 pneumotach and spirometer pod (ADInstruments, Dunedin, New Zealand). The fraction of expired O_2_ (F_E_O_2_), fraction of expired CO_2_ (F_E_CO_2_), P_ET_CO_2_, and P_ET_O_2_ were collected using a Gas Analyser, PowerLab 16/35 unit and LabChart 8 (ADInstruments, Dunedin, New Zealand). Inspired and expired gas concentrations were measured via a sampling tube from the facemask deadspace. Heart rate was recorded using a three-lead electrocardiogram and bioamplifier (ADInstruments, Dunedin, New Zealand). SpO_2_ was recorded using a clinical finger clip (Datex Ohmeda S5, Datex Ohmeda Inc., Wisconsin, USA).

Two questionnaires were administered during the protocol; the first was the 9-point Karolinska Sleepiness Score (KSS) to assess sleepiness, and the second was a visual analogue scale to assess 14 subjective symptoms (tiredness, coordination, dim vision, blurry vision, light-headedness, headache, perception of head swelling, nausea, faintness, boredom, restlessness, irritability, weakness, and worriedness). The subjective symptoms’ questionnaire consisted of a 250 mm line with “not at all” and “very” marking the left and right extremities, respectively. Participants responded by indicating the severity of each symptom anywhere on the appropriate line. The distance from the left end was measured and converted to a percentage. Each questionnaire was asked five times; before driving started (baseline) and after each driving task.

### fNIRS

Cerebral HbO, HbR, and total hemoglobin (HbT) were monitored using multi-channel functional near-infrared spectroscopy (Brainsight, Rogue Research Inc., Quebec, Canada). Sources and detectors were placed over the prefrontal and frontal cortices; these areas have been used in previous studies combining fNIRS with simulated driving (Bloomfield et al. 2021; Foy et al. 2016; Yoshino et al. 2013) and are associated with higher order cognitive processes, such as executive functions, as they are utilized in interpretation of visual input, planning, and execution. Two wavelengths of light (*λ*_1,2_ = 705 nm, 830 nm) were used at 20 mW of power. A total of 12 sources were used with 13 detectors to produce a total of 36 channels. All channels had an interoptode distance between 2.5 and 4 cm. An illustration of the optode layout is available at Supplementary Figure S1C (https://doi.org/10.6084/m9.figshare.17059727). The optode layout was registered to each participant’s head using a neuronavigation device (Brainsight, Rogue Research Inc., Quebec, Canada) and 3D position sensor (Polaris Vicra, NDI Medical); the array was then projected onto an MNI-152 brain template. fNIRS data were sampled at 10 Hz in Brainsight software (Brainsight, Rogue Research Inc., Quebec, Canada). An eyes-closed, resting baseline measure was taken before the first driving task began; participants were seated for at least 10 min before the baseline measure was recorded. Additional baseline measures were taken before and after the wash-in period for each condition; this accounted for potential baseline shifts between conditions.

### Pre-processing of fNIRS data

The raw signal from each channel was assessed for signal quality. A visual check was used to ensure that channels had clear pulsatile flow. Pre-processing was completed in NIRS toolbox (Santosa et al. 2018), with some steps completed using HomER functions (Huppert et al. 2009) via the Run_HOMER2 function within the toolbox.

Optical intensity data were first converted to optical density. Motion artefacts were detected automatically using the function ‘hmrMotionArtifactByChannel’ (tMotion = 0.5, tMask = 0.9, amp_thresh = 0.4, std_thresh = 4); baseline shifts were corrected using spline interpolation with a Savitzky–Golay filter (*p* = 0.99, FrameSize_sec = 5, ‘hmrMotionCorrectSpineSG’). Note that this correction is only applied to specific areas on a channel-by-channel basis. A low-pass filter (*f*_c_ = 0.09 Hz, ‘hmrBandpassFilt’) was applied to remove systemic physiological noise (Pinti et al. 2018). This cut-off value was selected to remove noise arising from breathing rate (~ 0.1 to 0.3 Hz), heart rate (~ 1 to 1.3 Hz), and Mayer waves (~ 0.1 Hz) while preserving low-frequency changes arising from the four gas conditions (Pinti et al. 2018; Yucel et al. 2021). Optical density data were converted to changes in HbO and HbR using the modified Beer–Lambert law (Cope et al. 1988). The differential path length factor was kept constant at 5.93 (Herold et al. 2017). Finally, HbT was calculated as the sum of HbO and HbR.

### Data analyses

Baseline values were averaged over approximately 2 min to obtain the mean value. Due to considerable variation in the calculated hemoglobin types, likely due to differences in hair and skin color, changes in hemoglobin type were normalized to room air baseline measures. The data from all channels were averaged to create a global average of the frontal cortex. While this method reduces spatial resolution, it provides clear interpretation of mean values while avoiding multiple channel-wise statistical tests. Channel-wise changes (in µmol) are presented in heat maps. Block averages were calculated for each condition to compare between-condition differences between normoxic and hypoxic baselines for the three hypoxic conditions. Linear mixed-effects models using Satterthwaite’s degrees of freedom method were applied for each of HbO, HbR, and HbT. fNIRS data were anatomically mapped using xjView (Cui et al. 2019) and presented visually using Surf Ice (University of South Carolina 2020). fNIRS data are presented as absolute changes (i.e., an increase or decrease in µM) for clarity.

### Statistical analysis

Statistical analyses were performed using Prism 9 (Graphpad Software Inc., San Diego, California, USA) and RStudio (R Core Team 2019). Most data sets were analyzed using a linear mixed-effects model conducted using the R package lmerTest (Kuznetsova et al. 2017) via RStudio (R Core Team 2019). The model was in the form variable ~ condition + 1 | subject, where variable is the variable of interest. condition had either 4 or 5 levels: baseline, normoxia (N), poikilocapnic hypoxia (PH), isocapnic hypoxia (IH), and hypercapnic hypoxia (HH). subject was the random effect from each participant. A *p* value of ≤ 0.05 was taken as statistically significant. If a significant difference was detected, post hoc pairwise comparisons using Tukey’s Honestly Significant Differences (Tukey’s HSD) were applied using the “emmeans” function from the emmeans package (Lenth 2021) to determine where between-condition differences lay. Count data (for example, number of speed exceedances) were analyzed using a general linear model with a Poisson distribution. The general linear model was applied using the same method as the linear mixed-effects model. If a significant difference was detected, Tukey’s HSD was applied using the function “glht” in the package multcomp (Hothorn et al. 2008). Due to considerable deviations from normality and many zero values, all subjective data were analyzed using Kruskal–Wallis tests, with post hoc Dunn’s multiple comparison tests. Unless otherwise stated, all data are presented as mean (SD). Error bars are 1 SD.

## Results

### Protocol adherence

One poikilocapnic, one isocapnic, and one hypercapnic trial were excluded due to mask leaks. One additional hypercapnic trial was omitted due to issues with nitrogen delivery, causing a return to normoxia. The hypoxic intervention was well tolerated, with only one participant stopping part way through an isocapnic trial (citing nausea; this trial was removed from analysis); however, the participant completed the remaining trials without issue. One participant did not complete the normoxic trial, citing unwillingness to continue; their remaining data were included in analysis. Therefore, the overall number of trials remaining were 20 normoxic, 20 poikilocapnic, 18 isocapnic, and 18 hypercapnic. Male and female participants exhibited similar physiological responses and there was insufficient power to explore effects of biological sex, so all participants were analyzed together.

### Physiological data

**Fig. 1:**
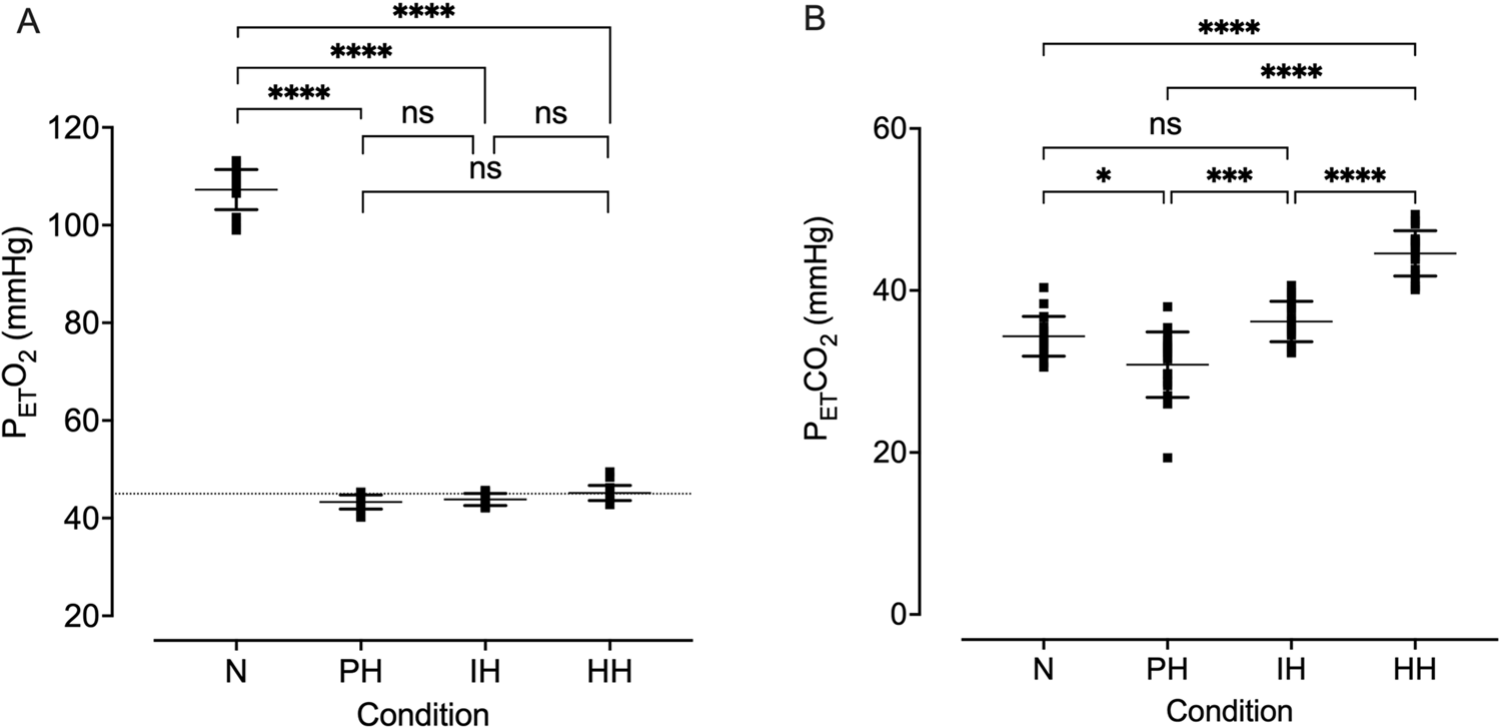
P_ET_O_2_ (**A**) and P_ET_CO_2_ (**B**) during simulated driving during normoxia (N), poikilocapnic hypoxia (PH), isocapnic hypoxia (IH), and hypercapnic hypoxia (HH). Individual data points are presented with the mean (horizontal bar) and SD (error bars). Horizontal dotted line indicates the desired P_ET_O_2_ level during hypoxia (45 mmHg). *n* = 20, 20, 18, and 18, for N, PH, IH, and HH, respectively. Data were analyzed using a linear mixed-effects model with Tukey HSD post hoc tests. *p ≤ 0.05; **p ≤ 0.01; ***p ≤ 0.001; ****p ≤ 0.0001

**Fig. 2:**
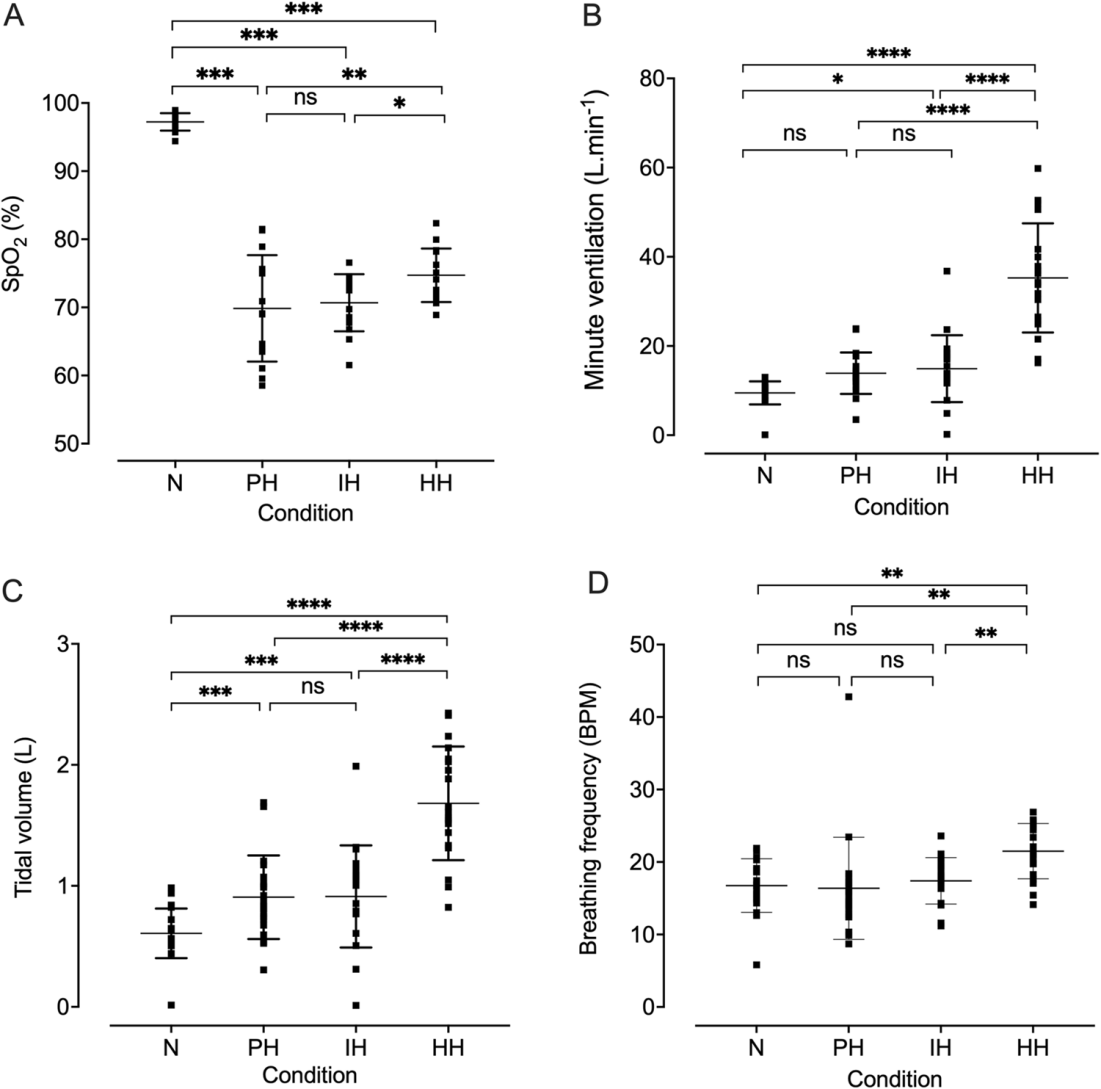
SpO_2_ (**A**), minute ventilation (**B**), tidal volume (**C**), and breathing frequency (**D**) during simulated driving during normoxia (N), poikilocapnic hypoxia (PH), isocapnic hypoxia (IH), and hypercapnic hypoxia (HH). Individual data points are presented with the mean (horizontal bar) and SD (error bars). *n* = 20, 20, 18, and 18, for N, PH, IH, and HH, respectively. Data were analyzed using a linear mixed-effects model with Tukey HSD post hoc tests. *p ≤ 0.05, **p ≤ 0.01, ***p ≤ 0.001, ****p ≤ 0.0001

The gas clamping method was successful in maintaining the desired P_ET_O_2_ value of 45 mmHg and, as expected, all hypoxic conditions reduced P_ET_O_2_ and SpO_2_ compared to normoxia (all *p* < 0.0001; Figs. 1A, 2A). SpO_2_ was lower in both isocapnic and poikilocapnic hypoxia compared to hypercapnic hypoxia (mean difference = 3.7% and 5.0%, respectively). P_ET_CO_2_ during isocapnic hypoxia did not differ significantly from normoxia (mean difference = 1.8 mmHg, *p* = 0.068, Fig. 1B). P_ET_CO_2_ during poikilocapnic hypoxia was lower than during normoxia and isocapnic hypoxia (*p* < 0.0001). P_ET_CO_2_ during the hypercapnic condition was greater than all other conditions (all *p* < 0.0001). Compared to normoxia, minute ventilation was greater in the isocapnic (*p* = 0.0162) and hypercapnic (*p* < 0.0001) conditions but was not significantly altered by poikilocapnic hypoxia (*p* = 0.0644). Minute ventilation was greater in hypercapnic hypoxia compared to the poikilocapnic and isocapnic conditions (both *p* < 0.001); the latter two conditions evoked similar responses (Fig. 2B). All hypoxic conditions increased tidal volume compared to baseline (*p* < 0.001); additionally, tidal volume was greater in hypercapnic hypoxia than both poikilocapnic and isocapnic hypoxia (both *p* < 0.0001, Fig. 2C). Only the hypercapnic condition modified breathing frequency (*p* ≤ 0.01 vs all other conditions, Fig. 2D). Heart rate increased in all hypoxic conditions compared to normoxia (all *p* ≤ 0.0001), but there were no differences between hypoxic conditions (71 (12), 89 (11), 88 (14) and 87 (16) beats.min^−1^, for normoxic, poikilocapnic, isocapnic, and hypercapnic conditions, respectively; all *p* > 0.05).

### Driving performance

**Fig. 3:**
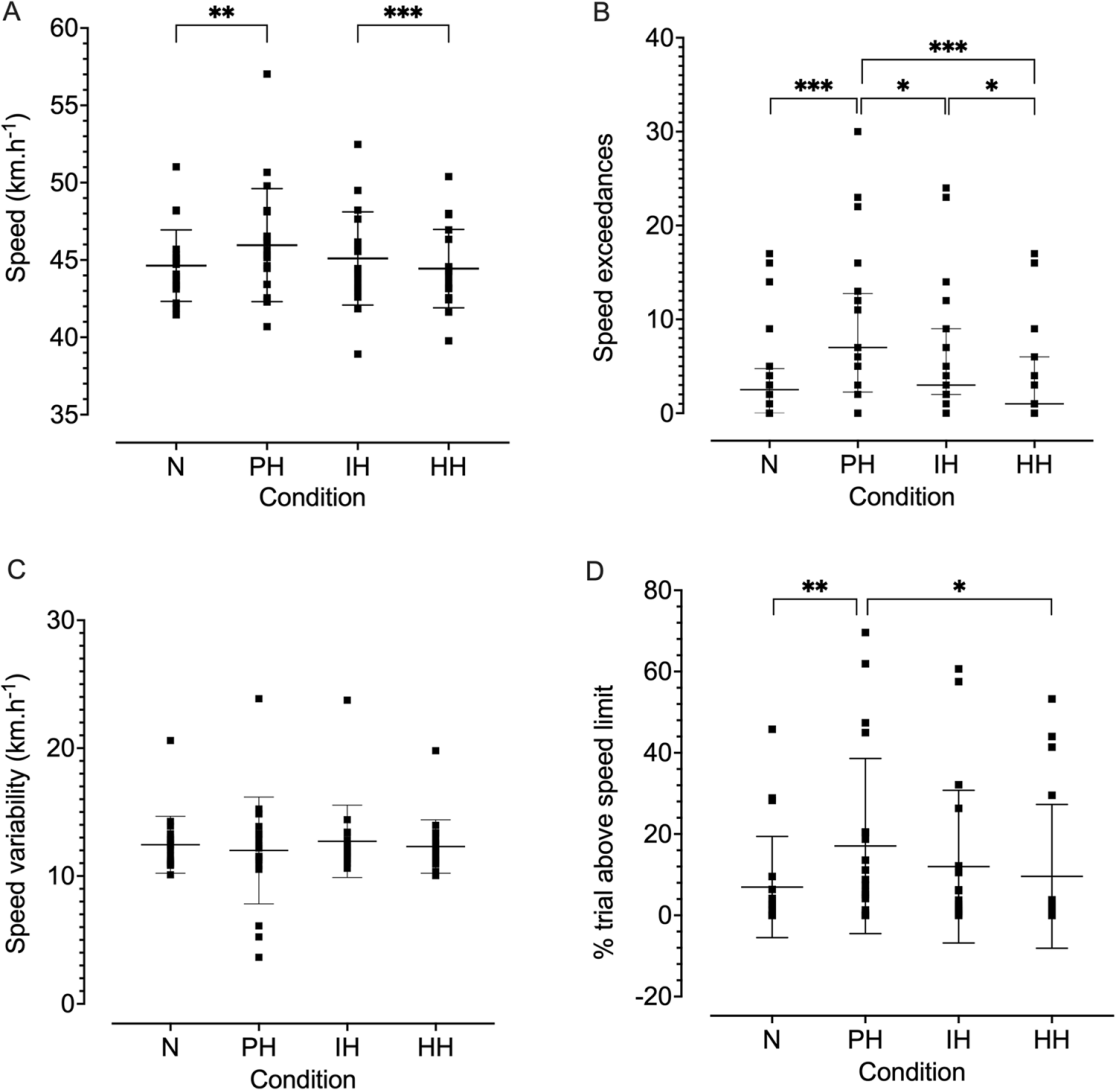
Vehicle speed (**A**), number of speed exceedances (**B**), speed variability (**C**), and percentage above the speed limit (**D**) during normoxia (N), poikilocapnic hypoxia (PH), isocapnic hypoxia (IH), and hypercapnic hypoxia (HH). Data are presented with as mean with SD error bars (**A**, **C**, **D**) and were analyzed using a linear mixed-effects model; data in **B** are presented as the median with interquartile range error bars and were analyzed using a general linear model. *n* = 20, 20, 18, and 18, for N, PH, IH, and HH, respectively. *p ≤ 0.05; **p ≤ 0.01; ***p ≤ 0.001; ****p ≤ 0.0001

Mean vehicle speed during poikilocapnic hypoxia was greater than during both the hypercapnic (*p* = 0.0002) and normoxic (*p* = 0.0096) conditions (Fig. 3A). The number of speed exceedances was greater in the isocapnic than hypercapnic condition (*p* = 0.0277), and greater in poikilocapnic hypoxia compared to the hypercapnic (*p* < 0.001), isocapnic (*p* = 0.0115), and normoxic (*p* < 0.001) conditions (Fig. 3B). Speed variability was unaffected by condition (*p* = 0.66), as were lane position (*p* = 0.74) and variability of lane position (*p* = 0.08) (Fig. 3C). The percentage of the trial spent above the speed limit (Fig. 3D) was greater in the poikilocapnic than normoxic (*p* = 0.0027) and hypercapnic (*p* = 0.0188) conditions. Two road edge excursions were recorded, both during poikilocapnic hypoxia. No center line crossings were recorded. One pedestrian was hit, during normoxia. No statistical tests were performed on these data due to the low counts.

### Cerebral oxygenation

Due to technical issues with the fNIRS machine (unrelated to the protocol), data were only recorded for 17 participants (~ 81%). The number of participants included for analysis in the normoxic, poikilocapnic, isocapnic, and hypercapnic conditions was 16, 16, 15, and 15, respectively. fNIRS data are presented as mean (95% confidence interval).

**Fig. 4:**
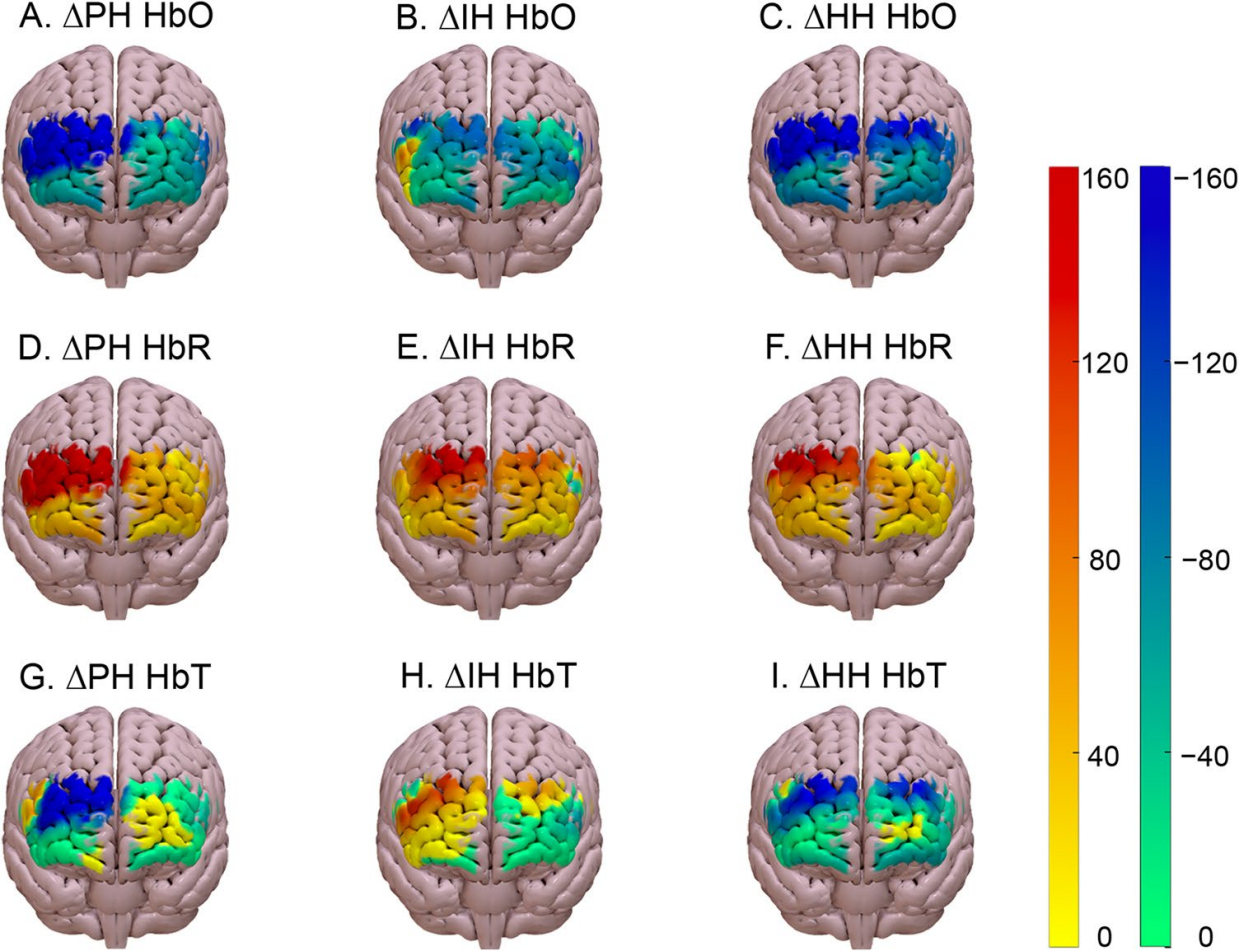
Changes in steady-state oxyhemoglobin (**A**–**C**), deoxyhemoglobin (**D–F**), and total hemoglobin (**G–I**) in the frontal cortex during poikilocapnic hypoxia (PH), isocapnic hypoxia (IH), and hypercapnic hypoxia (HH). Absolute changes from normoxic to hypoxic baseline for each hemoglobin type are shown above each panel. Progression from yellow to red indicates a larger absolute increase (µM); progression from green to blue indicates a larger absolute decrease (µM). The mean number of participants included in the analysis was 16, 16, 15, and 15, during N, PH, IH, and HH, respectively

Absolute channel-wise changes in baseline HbO, HbR, and HbT from normoxia during poikilocapnic, isocapnic, and hypercapnic conditions are shown in Fig. 4 and summed data are displayed in Table 1. HbO decreased and HbR increased in all hypoxic conditions. HbT was not significantly altered by any hypoxic condition (i.e., all confidence intervals encompassed zero). There were no between-condition differences for any hemoglobin type; for more information on between-condition effect sizes, see Supplementary Fig. S2 (available at https://doi.org/10.6084/m9.figshare.20195144.v1).

**Table 1:** Effects of gas manipulation on oxyhaemoglobin (HbO), deoxyhaemoglobin (HbT), and total hemoglobin (HbT) in frontal cortex at baseline

	HbO (µmol)	HbR (µmol)	HbT (µmol)
PH	− 225 [− 344, − 107]	159 [82, 235]	− 38 [− 122, 46]
IH	− 138 [− 192, − 84]	124 [79, 168]	− 33 [− 75, 9]
HH	− 164 [− 245, − 82]	100 [57, 142]	8 [− 64, 81]

PH, poikilocapnic hypoxic; IH, isocapnic hypoxia; HH, hypercapnic hypoxia. *n* = 16, 15, and 15, for PH, IH, and HH, respectively. Mean changes from resting normoxic baseline (in µmol) are shown with 95% confidence intervals. Conditions were compared using a linear mixed-effects model. Values showing a significant change from baseline are in bold

### Subjective symptoms

There were no differences between any hypoxic conditions for any symptom (Table 1). Compared to normoxia, perceived coordination was worse in the isocapnic (*p* = 0.001) and poikilocapnic (*p* = 0.002) conditions. Blurry vision and dim vision were worse in poikilocapnic hypoxia compared to baseline (*p* = 0.0117 and *p* = 0.0035, respectively). All hypoxic conditions worsened the feeling of light-headedness, compared to both baseline and normoxia (all *p* ≤ 0.05). Compared to baseline, the headache symptom was worsened by hypercapnic hypoxia (*p* = 0.0087). All hypoxic conditions worsened the swollen head and faintness symptoms compared to baseline (all *p* ≤ 0.05). Perceived weakness was greater in the poikilocapnic and isocapnic conditions compared to both baseline (both *p* ≤ 0.05); weakness was greater in the poikilocapnic than normoxic condition (*p* = 0.0143). Subjective sleepiness was greater in the poikilocapnic condition compared to baseline (*p* = 0.0056) and in the isocapnic condition compared to baseline (*p* = 0.026).

## Discussion

We aimed to determine the independent effect of arterial CO_2_ concentration on complex task performance (i.e., simulated driving) during severe oxygen deprivation. The major novel finding of the present study is that poikilocapnic hypoxia caused a two-fold increase in speed exceedances and a greater mean vehicle speed compared to normoxia. In contrast, mean vehicle speed and the number of speed exceedances in the hypercapnic and isocapnic conditions did not differ from normoxia. In support of our original hypothesis, these findings demonstrate the neuroprotective role of CO_2_ on complex psychomotor task performance during severe hypoxia. These results are pertinent, because speeding is an example of a dangerous behaviour, strongly linked to fatalities (Ministry of Transport 2021; Patterson et al. 2000), that appears to emerge readily with oxygen deprivation during a familiar task.

We successfully induced hypoxia by clamping P_ET_O_2_ at ~ 45 mmHg, while P_ET_CO_2_ was manipulated, such that it was either poikilocapnic [PH; 31 (4.0) mmHg], isocapnic [IH; 36 (2.5) mmHg], or hypercapnic [HH; 45 (2.8) mmHg). Frontal cortex HbO decreased and HbR increased in all hypoxic conditions (Fig. 4, Table 1), confirming that the hypoxic intervention impaired cortical oxygenation in areas previously identified as being up-regulated during simulated driving (Bloomfield et al. 2021; Foy et al. 2016). Such alterations in systemic and cerebral oxygenation have previously been associated with decrements in cognitive function (McMorris et al. 2017). We initially hypothesized that supplementation of CO_2_ during hypoxia would protect against reductions in the performance of this complex cognitive task, and indeed, performance impairments were not observed in the isocapnic and hypercapnic conditions. This hypothesis was predicated on the understanding that CO_2_ is a powerful vasodilator of the cerebral blood vessels and that vasoconstriction, secondary to the hyperventilation-mediated reduction in PaCO_2_, could impair oxygen availability and compromise cognitive function during poikilocapnic hypoxia (Ainslie and Poulin 2004; Shapiro et al. 1970). Indeed, the mean decline in P_ET_CO_2_ (3.5 mmHg, Fig. 1B) during poikilocapnic hypoxia would be expected to cause a considerable reduction in CBF (Battisti-Charbonney et al. 2011; Kety and Schmidt 1948). Moreover, the concomitant lowering of PaO_2_ and increase in PaCO_2_ during hypercapnic hypoxia would be expected to increase CBF and CBV (Ainslie and Poulin 2004), protecting cognitive function. However, HbT, an fNIRS derived index of cerebral blood volume (Yoshino et al. 2013), remained unchanged by hypoxia and did not differ across the three hypoxic conditions (Table 1, Fig. 4G–I). Furthermore, the similarity of hemodynamic responses under hypoxic conditions suggests that the observed overall reductions in cerebral oxygenation are not strongly linked to the observed differences in performance outcomes during hypoxia. It is likely that mechanisms other than reduced cerebral oxygen availability play an important role in cognitive function during hypoxia. Although respiratory rate was greater in the hypercapnic condition, previous research shows that ventilation does not affect cerebral blood velocity during isocapnic hypoxia (AlSalahi et al. 2021). However, it is possible there is a direct effect of CO_2_ on neuronal function; Bain et al. (Bain et al. 2016) reported that severe hypercapnic hypoxia reduced the cerebral metabolic rate of oxygen, which may have reduced oxygen consumption in less critical brain areas.

Both poikilocapnic and isocapnic hypoxia increased sleepiness, compared to baseline (Table 2), in accordance with a previous study showing that a simulated altitude of 4300 m (sea level equivalent F_i_O_2_ = 12.3%) increased subjective fatigue in helicopter pilots (Bouak et al. 2018). We showed that poikilocapnic hypoxia caused considerable worsening of the dim and blurry vision symptoms (Table 2); in contrast, previous research conducted in aircrew (Woodrow et al. 2011) and non-aircrew (Blacker and McHail 2021) reported that these were uncommon symptoms. Hypercapnia has traditionally been associated with worse subjective feelings during normoxia, due in part to the relationship between respiratory rate and feelings of panic (Colasanti et al. 2008, 2007). However, hypercapnia has previously been shown to preserve visual function during hypoxia (Stepanek et al. 2014). In our paradigm, the dim and blurry vision symptoms were worsened by poikilocapnic hypoxia but were unchanged by the isocapnic and hypercapnic conditions. The worse symptoms during the poikilocapnic condition likely contributed to the observed impairments in driving performance, which were not seen during isocapnic or hypercapnic hypoxia.

**Table 2:** Subjective symptoms

Symptom	B	N	PH	IH	HH
Tired	21.2 (31.6)	28.8 (48.1)	41.2 (38.8)	38.8 (43.7)	33.2 (38.0)
Coordination off	1.2 (10.4)^b,c^	2.2 (11.2)^b,c^	20.4 (42.0)	22.6 (27.8)	12.4 (17.6)
Dim vision	0.0 (1.4)^b^	0.4 (5.4)	15.2 (60.8)	11.2 (30.4)	9.6 (21.2)
Blurry vision	0.0 (3.2)^b^	0.0 (8.8)	18.4 (61.6)	10.4 (33.4)	12.8 (28.8)
Light-headedness	0.0 (0.2)^b,c,d^	1.6 (6.9)^b,c,d^	40.0 (42.0)	36.2 (50.8)	23.2 (39.6)
Headache	0.0 (0.8)^d^	0.0 (7.2)	15.6 (35.2)	19.6 (44.2)	13.6 (44.4)
Swollen head	0.0 (0.2)^b,c,d^	0.0 (5.7)	11.6 (30.8)	10.0 (37.1)	9.2 (30.8)
Weak	0.0 (5.8)^b,c^	2.8 (7.2)^b^	14.8 (34.0)	10.0 (32.5)	10.0 (18.0)
Bored	0.0 (4.0)	4.0 (27.8)	6.0 (24.8)	7.2 (27.1)	0.0 (10.4)
Sick	0.0 (0.6)	0.0 (1.8)	0.8 (10.4)	0.0 (3.4)	0.0 (7.6)
Faint	0.0 (0.6)^b,c,d^	0.0 (4.8)	8.8 (30.4)	10.8 (23.0)	5.2 (20.8)
Irritable	0.0 (1.4)	0.0 (3.1)	0.0 (6.8)	0.0 (5.7)	0.8 (8.8)
Restless	0.0 (8.2)	0.0 (7.6)	12.0 (33.2)	3.4 (17.7)	4.8 (20.0)
Worried	0.0 (3.6)	0.0 (2.0)	2.0 (13.2)	0.0 (12.1)	0.0 (6.8)
KSS	3.0 (3.0)^b,c^	5.0 (3.0)	6.0 (2.0)	6.0 (3.0)	5.0 (2.3)

*B* baseline, *N* normoxia, *PH* poikilocapnic hypoxia, *IH* isocapnic hypoxia, *HH* hypercapnic hypoxia. *n* = 21, 20, 18, 15, and 16, for B, N, PH, IH, and HH, respectively. Data are presented as median (interquartile range) and were assessed via Kruskal–Wallis tests with Dunn’s post hoc comparisons

^a^*p* ≤ 0.05 vs N

^b^*p* ≤ 0.05 vs PH

^c^*p* ≤ 0.05 vs IH

^d^*p* ≤ 0.05 vs HH

Although it seems intuitive that higher order cognitive functions, such as those involved in complex tasks like driving, should be more readily affected by hypoxia than lower order cognitive functions, this assumption is not universally supported (McMorris et al. 2017). More complex tasks combining multiple neurocognitive domains may initiate a greater hemodynamic response, thereby offsetting declines in cognitive function induced by low oxygen availability. While simplistic, many of the cognitive tasks utilized in previous studies are likely to be unfamiliar to participants and may be more sensitive to changes in oxygen availability. In contrast, as all participants were very familiar with driving, they may have been able to complete the task with a reduced cognitive demand, compared to a novel cognitive task, due to greater task automation (Paxion et al. 2014).

Interestingly, variables associated with control of vehicle position, such as variability of lane position, were unchanged by hypoxia. This was surprising as previous studies in normoxia have shown these measures to be sensitive to changes in cognitive function induced by fatigue (Contardi et al. 2004; Gastaldi et al. 2014) and alcohol (Mets et al. 2011). It is possible that the relatively short duration of the hypoxic stress was a contributing factor. Although the severity of the hypoxic intervention was like that used in the previous studies (Friend et al. 2019; Turner et al. 2015a, b; Turner et al. 2015a, b), we only used a 3-min wash-in period, while Turner et al. (2015a, b) utilized a 30-min wash-in period. However, Stepanek et al. (2014) reported impairments in King-Devick performance using a 3-min hypoxic exposure, with slightly higher SpO_2_ values. In sum, task familiarity, as well as duration and severity of exposure, may affect the magnitude of the hypoxia-induced impairment in cognitive function.

### Experimental considerations

Cerebral blood velocity or flow was not measured, for example using Doppler ultrasound. However, neither extra- nor intra-cranial ultrasound measures can determine regional perfusion at the cortical level, which means that any redistribution of blood flow above the cerebral arteries cannot be determined. We were unable to measure arterial pH or changes in bicarbonate buffering, which can differ between participants. It is possible there was some contamination of the fNIRS signal due to skin blood flow (Yucel et al. 2021); however, we attempted to minimize this issue by ensuring that all channels had the optimal interoptode distance for measuring cortical oxygenation.

### Conclusion

We measured simulated driving performance and cerebral oxygenation during severe poikilocapnic, isocapnic, and hypercapnic hypoxia. Vehicle speed regulation was impaired by poikilocapnic hypoxia, likely due to the effects of acute respiratory alkalosis, but were largely unaffected when CO_2_ was controlled during isocapnic and hypercapnic hypoxia. These findings indicate that CO_2_ can exert a protective effect on real-world task performance and brain energetics during severe hypoxia. However, this effect does not seem to be due to alterations in cortical oxygenation, as assessed by fNIRS. Further research into the independent effects of task familiarity and duration of hypoxic exposure on cognitive function should be conducted.

## Supplementary Information

Below is the link to the electronic supplementary material.

Supplementary Fig. S1. Driving simulator set up (A), driving scenario (B) and optode montage (C). Optode montage is mapped using the 10-20 system. Channels are denoted by blue lines, sources are red dots, and detectors are blue dots (TIF 2056 KB)

Supplementary Fig. S2. Between-condition effect sizes for oxyhaemoglobin (A-C), deoxyhemoglobin (D-F), and total hemoglobin (G-I) in the frontal cortex during poikilocapnic hypoxia (PH), isocapnic hypoxia (IH) and hypercapnic hypoxia (HH). Cohen's d effect sizes for each comparison are displayed as heat maps for each hemoglobin type. Comparisons are shown above each panel. Progression from yellow to red indicates a larger positive effect size; progression from green to blue indicates a larger negative effect size. The mean number of participants included in the analysis was 16, 16, 15, and 15, during N, PH, IH, and HH, respectively (TIF 1860 KB)

## Data Availability

The data supporting the findings of this study are available from the authors upon reasonable request.

## Abbreviations

CBF: Cerebral blood flow
CBV: Cerebral blood volume
CO_2_: Carbon dioxide
fNIRS: Functional near-infrared spectroscopy
HH: Hypercapnic hypoxia
IH: Isocapnic hypoxia
N: Normoxia
N_2_: Nitrogen
PH: Poikilocapnic hypoxia
O_2_: Oxygen

## References

[CR1] Ainslie PN, Poulin MJ (2004). Ventilatory, cerebrovascular, and cardiovascular interactions in acute hypoxia: regulation by carbon dioxide. J Appl Physiol.

[CR38] AlSalahi SE, Junejo RT, Bradley C, Balanos GM, Siebenmann C, Fisher JP (2021) The middle cerebral artery blood velocity response to acute normobaric hypoxia occurs independently of changes in ventilation in humans. Exp Physiol 106(4):861–867. 10.1113/EP089127 10.1113/EP089127 33527604

[CR2] Bain AR, Ainslie PN, Hoiland RL, Barak OF, Cavar M, Drvis I, Stembridge M, MacLeod DM, Bailey DM, Dujic Z, MacLeod DB (2016). Cerebral oxidative metabolism is decreased with extreme apnoea in humans; impact of hypercapnia. J Physiol.

[CR3] Battisti-Charbonney A, Fisher J, Duffin J (2011). The cerebrovascular response to carbon dioxide in humans. J Physiol.

[CR4] Blacker KJ, McHail DG (2021). Time course of recovery from acute hypoxia exposure as measured by vigilance and event-related potentials. Physiol Behav.

[CR5] Bloomfield PM, Green H, Gant N (2021). Cerebral haemodynamics during simulated driving: changes in workload are detectable with functional near infrared spectroscopy. PLoS ONE.

[CR6] Bouak F, Vartanian O, Hofer K, Cheung B (2018). Acute mild hypoxic hypoxia effects on cognitive and simulated aircraft pilot performance. Aerosp Med Hum Perform.

[CR7] Colasanti A, van Diest R, Salamon E, Schruers K, Griez EJ (2007). Carbon dioxide inhalation induces dose-dependent and age-related negative affectivity. PLoS ONE.

[CR8] Colasanti A, Salamon E, Schruers K, van Diest R, van Duinen M, Griez EJ (2008). Carbon dioxide-induced emotion and respiratory symptoms in healthy volunteers. Neuropsychopharmacology.

[CR9] Contardi S, Pizza F, Sancisi E, Mondini S, Cirignotta F (2004). Reliability of a driving simulation task for evaluation of sleepiness. Brain Res Bull.

[CR10] Cope M, Delpy DT, Reynolds EOR, Wray S, Wyatt J, van der Zee P (1988). Methods of quantitating cerebral near infrared spectroscopy data. Adv Exp Med Biol.

[CR11] Cui X, Li J, Song X, Ma Z (2019) xjView. https://www.alivelearn.net/xjview. Acessed Aug 2021

[CR12] Foy HJ, Runham P, Chapman P (2016). Prefrontal cortex activation and young driver behaviour: a fNIRS study. PLoS ONE.

[CR13] Friend AT, Balanos GM, Lucas SJE (2019). Isolating the independent effects of hypoxia and hyperventilation-induced hypocapnia on cerebral haemodynamics and cognitive function. Exp Physiol.

[CR14] Gastaldi M, Rossi R, Gecchele G (2014). Effects of driver task-related fatigue on driving performance. Procedia Soc Behav Sci.

[CR15] Green HM, Borges VM, Connell CWJ, Newcombe D, Thompson B, Gant N (2019). Reliability of oculomotor kinematics, visual attention, and vehicle operation in driving simulation. Investig Ophthalmol vis Sci.

[CR16] Herold F, Wiegel P, Scholkmann F, Thiers A, Hamacher D, Schega L (2017). Functional near-infrared spectroscopy in movement science: a systematic review on cortical activity in postural and walking tasks. Neurophotonics.

[CR17] Hothorn T, Bretz F, Westfall P (2008). Simultaneous inference in general parametric models. Biom J.

[CR18] Huppert TJ, Diamond SG, Franceschini MA, Boas DA (2009). HomER: a review of time-series analysis methods for near-infrared spectroscopy of the brain. Appl Opt.

[CR19] Kety SS, Schmidt CF (1948). The effects of altered arterial tensions of carbon dioxide and oxygen on cerebral blood flow and cerebral oxygen consumption of normal young men. J Clin Investig.

[CR20] Kuznetsova A, Brockhoff PB, Christensen RHB (2017) lmerTest Package: tests in linear mixed effects models. J Stat Softw 82(13):1–26. 10.18637/jss.v082.i13

[CR21] Leacy JK, Day TA, O'Halloran KD (2019). Is alkalosis the dominant factor in hypoxia-induced cognitive dysfunction?. Exp Physiol.

[CR22] Lenth RV (2021) emmeans: estimated marginal means, aka least-squares means. In: (Version R package version 1.5.4.). https://CRAN.R-project.org/package=emmeans. Accessed Aug 2021

[CR23] McMorris T, Hale BJ, Barwood M, Costello J, Corbett J (2017). Effect of acute hypoxia on cognition: a systematic review and meta-regression analysis. Neurosci Biobehav Rev.

[CR24] Meldrum NU, Roughton FJW (1933). Carbonic anhydrase: its preparation and properties. J Physiol.

[CR25] Mets MA, Kuipers E, de SenerpontDomis LM, Leenders M, Olivier B, Verster JC (2011). Effects of alcohol on highway driving in the STISIM driving simulator. Hum Psychopharmacol.

[CR26] Ministry of Transport (2021) Safety—annual statistics. https://www.transport.govt.nz/statistics-and-insights/safety-annual-statistics/sheet/speed. Accessed Sept 2021

[CR27] Ogoh S (2015). Cerebral blood flow regulation during hypoxia. Exp Physiol.

[CR28] Ogoh S (2019). Interaction between the respiratory system and cerebral blood flow regulation. J Appl Physiol.

[CR29] Ogoh S, Sato K, Nakahara H, Okazaki K, Subudhi AW, Miyamoto T (2013). Effect of acute hypoxia on blood flow in vertebral and internal carotid arteries. Exp Physiol.

[CR30] Patterson TL, Frith WJ, Small MW (2000) Down with speed: a review of the literature, and the impact of speed on New Zealanders

[CR31] Paxion J, Galy E, Berthelon C (2014). Mental workload and driving. Front Psychol.

[CR32] Pinti P, Scholkmann F, Hamilton A, Burgess P, Tachtsidis I (2018). Current status and issues regarding pre-processing of fNIRS neuroimaging data: an investigation of diverse signal filtering methods within a general linear model framework. Front Hum Neurosci.

[CR33] R Core Team (2019) R: a language and environment for statistical computing. In: (Version 1.2.5033) https://www.R-project.org/. Accessed Aug 2021

[CR34] Santosa H, Zhai X, Fishburn F, Huppert T (2018). The NIRS brain AnalyzIR toolbox. Algorithms.

[CR35] Shapiro W, Wasserman AJ, Baker JP, Patterson JL (1970). Cerebrovascular response to acute hypocapnic and eucapnic hypoxia in normal man. J Clin Investig.

[CR36] Shaw DM, Cabre G, Gant N (2021). Hypoxic hypoxia and brain function in military aviation: basic physiology and applied perspectives. Front Physiol.

[CR37] Stepanek J, Pradhan GN, Cocco D, Smith BE, Bartlett J, Studer M, Kuhn F, Cevette MJ (2014). Acute hypoxic hypoxia and isocapnic hypoxia effects on oculometric features. Aviat Space Environ Med.

[CR39] Turner CE, Barker-Collo SL, Connell CJ, Gant N (2015). Acute hypoxic gas breathing severely impairs cognition and task learning in humans. Physiol Behav.

[CR40] Turner CE, Byblow WD, Gant N (2015). Creatine supplementation enhances corticomotor excitability and cognitive performance during oxygen deprivation. J Neurosci.

[CR41] University of South Carolina (2020) Surf ice. https://www.nitrc.org/projects/surfice/. Accessed Sept 2021

[CR42] Woodrow AD, Webb JT, Wier GS (2011). Recollection of hypoxia symptoms between training events. Aviat Space Environ Med.

[CR43] Yoshino K, Oka N, Yamamoto K, Takahashi H, Kato T (2013). Functional brain imaging using near-infrared spectroscopy during actual driving on an expressway. Front Hum Neurosci.

[CR44] Yucel MA, Luhmann AV, Scholkmann F, Gervain J, Dan I, Ayaz H, Boas D, Cooper RJ, Culver J, Elwell CE, Eggebrecht A, Franceschini MA, Grova C, Homae F, Lesage F, Obrig H, Tachtsidis I, Tak S, Tong Y, Wolf M (2021). Best practices for fNIRS publications. Neurophotonics.

