# Supplementary figures and images for: Carbon dioxide protects simulated driving performance during severe hypoxia
Source: Eur J Appl Physiol. 2023 Mar 23;123(7):1583–93. doi: 10.1007/s00421-023-05151-1 (PMC10276124; doi:10.1007/s00421-023-05151-1)

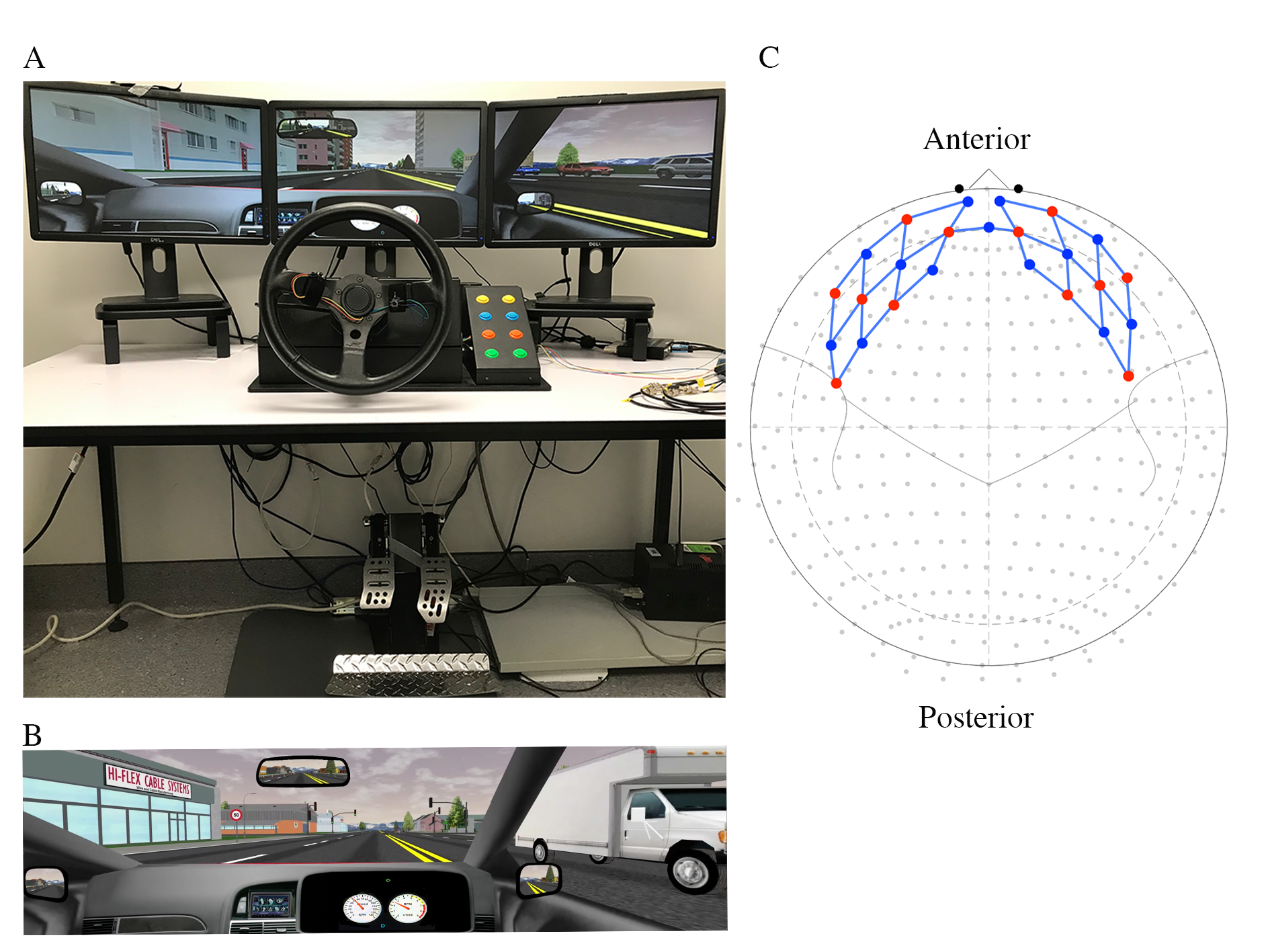

Supplement: Supplementary file 1 — Supplementary Fig. S1. Driving simulator set up (A), driving scenario (B) and optode montage (C). Optode montage is mapped using the 10-20 system. Channels are denoted by blue lines, sources are red dots, and detectors are blue dots (TIF 2056 KB) [file 421_2023_5151_MOESM1_ESM.tif]

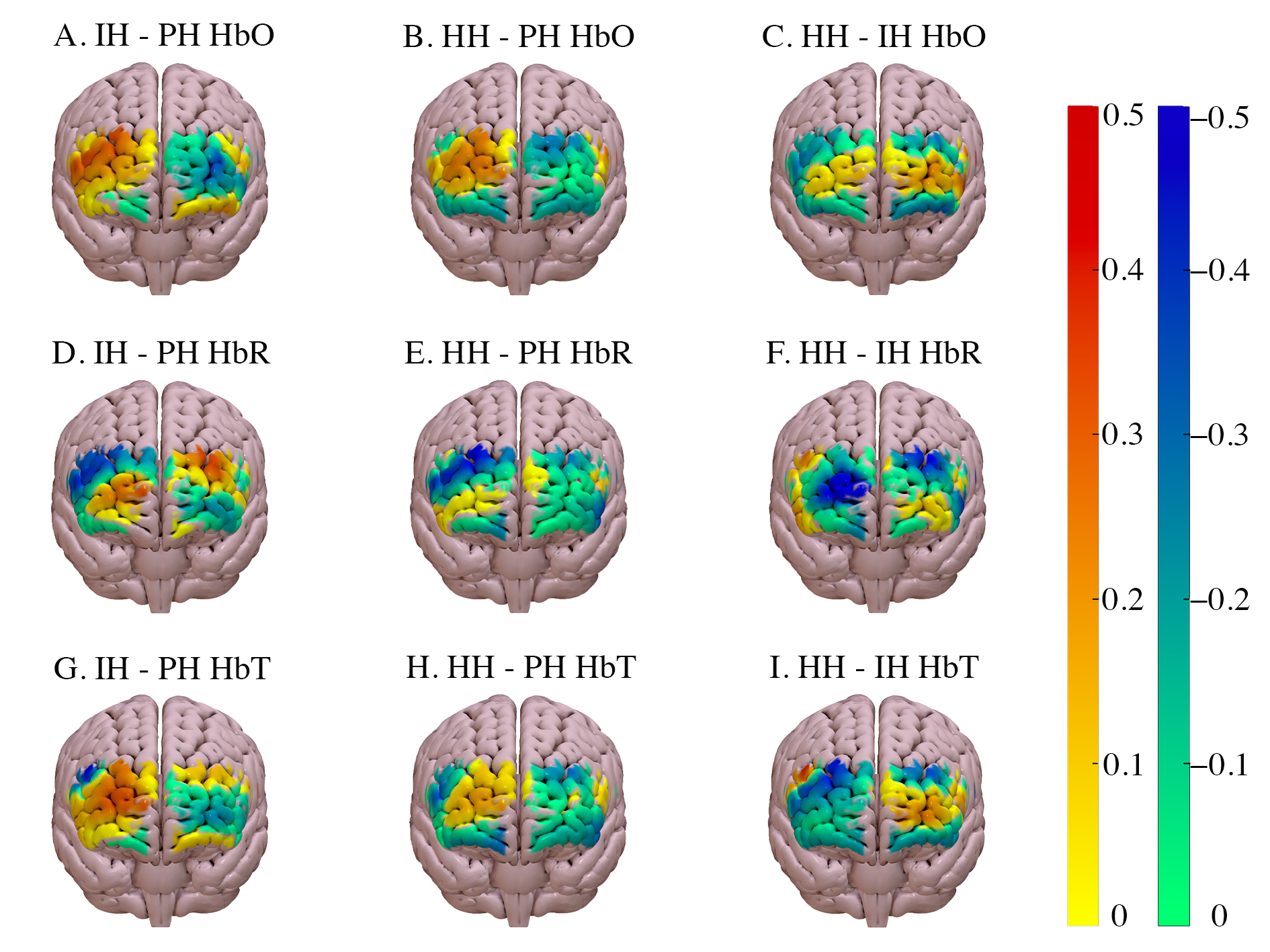

Supplement: Supplementary file 2 — Supplementary Fig. S2. Between-condition effect sizes for oxyhaemoglobin (A-C), deoxyhemoglobin (D-F), and total hemoglobin (G-I) in the frontal cortex during poikilocapnic hypoxia (PH), isocapnic hypoxia (IH) and hypercapnic hypoxia (HH). Cohen's d effect sizes for each comparison are displayed as heat maps for each hemoglobin type. Comparisons are shown above each panel. Progression from yellow to red indicates a larger positive effect size; progression from green to blue indicates a larger negative effect size. The mean number of participants included in the analysis was 16, 16, 15, and 15, during N, PH, IH, and HH, respectively (TIF 1860 KB) [file 421_2023_5151_MOESM2_ESM.tif]
